# Resistivity Technique for the Evaluation of the Integrity of Buccal and Esophageal Epithelium Mucosa for In Vitro Permeation Studies: Swine Buccal and Esophageal Mucosa Barrier Models

**DOI:** 10.3390/pharmaceutics13050643

**Published:** 2021-04-30

**Authors:** Jaiza Samara Macena de Araújo, Maria Cristina Volpato, Bruno Vilela Muniz, Gabriela Gama Augusto Xavier, Claudia Cristina Maia Martinelli, Renata Fonseca Vianna Lopez, Francisco Carlos Groppo, Michelle Franz-Montan

**Affiliations:** 1 Department of Biosciences, Piracicaba Dental School, University of Campinas-UNICAMP, Piracicaba 13414-903, São Paulo, Brazil; j181581@dac.unicamp.br (J.S.M.d.A.); volpato@fop.unicamp.br (M.C.V.); bruno.vilela@professor.fait.edu.br (B.V.M.); g168300@dac.unicamp.br (G.G.A.X.); claudia_marabesi@br.ajinomoto.com (C.C.M.M.); fcgroppo@unicamp.br (F.C.G.); 2 School of Pharmaceutical Sciences of Ribeirão Preto, University of São Paulo-USP, Ribeirão Preto 14040-903, São Paulo, Brazil; rvianna@fcfrp.usp.br

**Keywords:** membrane resistivity, esophageal epithelium, buccal epithelium, mucosal drug delivery, in vitro models, transbuccal drug delivery

## Abstract

Permeation assays are important for the development of topical formulations applied on buccal mucosa. Swine buccal and esophageal epithelia are usually used as barriers for these assays, while frozen epithelia have been used to optimize the experimental setup. However, there is no consensus on these methods. In transdermal studies, barrier integrity has been evaluated by measuring electrical resistance (ER) across the skin, which has been demonstrated to be a simple, fast, safe, and cost-effective method. Therefore, the aims here were to investigate whether ER might also be an effective method to evaluate buccal and esophageal epithelium mucosa integrity for in vitro permeation studies, and to establish a cut-off ER value for each epithelium mucosa model. We further investigated whether buccal epithelium could be substituted by esophageal epithelium in transbuccal permeation studies, and whether their permeability and integrity were affected by freezing at −20 °C for 3 weeks. Fresh and frozen swine buccal and esophageal epithelia were mounted in Franz diffusion cells and were then submitted to ER measurement. Permeation assays were performed using lidocaine hydrochloride as a hydrophilic drug model. ER was shown to be a reliable method for evaluating esophageal and buccal epithelia. The esophageal epithelium presented higher permeability compared to the buccal epithelium. For both epithelia, freezing and storage led to decreased electrical resistivity and increased permeability. We conclude that ER may be safely used to confirm tissue integrity when it is equal to or above 3 kΩ for fresh esophageal mucosa, but not for buccal epithelium mucosa. However, the use of esophageal epithelium in in vitro transmucosal studies could overestimate the absorption of hydrophilic drugs. In addition, fresh samples are recommended for these experiments, especially when hydrophilic drugs are involved.

## 1. Introduction

Oral administration is one of the most convenient forms of drug delivery, due to the ease of ingestion and convenience for the patient. However, using this route, drugs might be altered due to first-pass metabolism and enzymatic degradation in the gastrointestinal tract. Topical administration through the buccal mucosa (inside the cheek) is a promising administration route. Transbuccal delivery overcomes the undesirable aspects of oral administration, increasing the compliance of patients since this pathway is non-invasive, offers simple application, and the medication may be easily removed in cases of adverse effects or allergies [1,2,3].

However, transbuccal topical administration has some disadvantages, such as the limited area available for absorption (200 cm^2^) [4,5] and relatively low permeability, since the physiological function of the buccal mucosa is to act as a barrier [6]. This may require the use of permeation enhancers or even drug delivery systems, in order to increase drug permeation [1,7,8,9,10]. Additionally, formulations for buccal delivery should have a palatable taste and appropriate mucoadhesive characteristics [9].

In vitro permeation assays are essential for evaluation of the performance and quality of newly designed formulations, considering the physicochemical characteristics of combinations of components and excipients tested during the development of topical formulations [11]. In addition, these assays can enable prediction of the absorption and bioavailability of new formulations, because they can simulate various complex biochemical arrangements and physical barriers, helping to predict in vivo effectiveness [3,12]. In vitro permeation assays are typically performed using vertical diffusion cells, where a barrier is installed between the donor and receptor compartments, with the amount of a drug from the formulation that crosses the barrier being quantified in the donor compartment.

For transbuccal or transmucosal formulations, human buccal tissues are not usually used, because they are difficult to obtain and approval by ethics committees is required. Swine esophageal and buccal epithelium mucosas have been demonstrated to have similar histological, structural, and compositional features as the corresponding human tissues [13,14]. Furthermore, swine tissues are easily obtained, since there is widespread slaughter of the animals for food consumption, enabling reduction of the use of animals in research [15,16,17,18].

Since mastication might damage the swine buccal epithelium, and only small areas of this mucosa are available, studies have proposed the use of esophageal mucosa as a buccal mucosa substitute, due to its similar histology and lipid composition, as well as simpler preparation [13,14]. In addition, the esophageal mucosa tissue is more convenient to prepare and has a larger viable area [13,14,19]. It has also been proposed that the epithelium could be frozen and stored, creating a tissue bank for future permeation assays [13]. However, there is no consensus regarding the interchangeability of these epithelia and the effect of freezing on permeability [19].

Verification of tissue integrity prior to permeation assays was not performed in most of the early studies involving buccal and esophageal barrier models [13,14,15,19,20]. The selection of undamaged tissues by visual inspection was described later [21]. However, this may not be sufficient to ensure tissue integrity.

Skin integrity is typically assessed prior to transdermal permeation assays. Among the different methods available, measuring electrical resistance (ER) across the skin has been used since the 1990s [22,23], and is considered to be simple, fast, safe, and cost-effective [24,25,26].

In 1991, De Vries and colleagues described the use of this method to evaluate the permeability of dermatome buccal mucosa samples (0.24–0.80 mm thickness), obtaining ER values varying between 1 and 2 kΩ/cm^2^ [27]. Later, ER was used to predict the integrity of esophageal epithelium mucosa [28]. It was found that the permeation of lidocaine and prilocaine was independent of ER for values between 2.8 and 12.7 kΩ/cm^2^, but increased dramatically for ER lower than 2.5 kΩ/cm^2^. Therefore, we hypothesized that ER could be used as a reliable method to select intact epithelium mucosa barriers for permeation studies.

The aims of the present study were to determine whether buccal and esophageal epithelium mucosa integrity can be predicted by ER for in vitro permeation studies, as well as to establish a cut-off ER value for each epithelium mucosa model, in order to predict barrier integrity. The possibility of substituting buccal epithelium by esophageal epithelium in transbuccal permeation studies was evaluated, together with assessment of the effect of freezing for 3 weeks at −20 °C on the permeability and integrity of the epithelia.

## 2. Material and Methods

### 2.1. Material

Lidocaine hydrochloride, acetonitrile, and ammonium hydroxide (HPLC grade) were purchased from Sigma-Aldrich (St Louis, MO, USA). Carbopol, propylene glycol, methylparaben, triethanolamine, and glycerin were obtained from Galena Química e Farmacêutica (Campinas, São Paulo, Brazil). Ultra-purified deionized water (Milli-Q system, Millipore Corporation, Billerica, MA, USA) was used for the preparation of aqueous solutions, including phosphate-buffered saline (PBS) (8 g/L of NaCl, 0.2 g/L of KCl, 1.44 g/L of Na_2_HPO_4_, and 0.24 g/L of KH_2_PO_4_; 1×, pH 7.4) produced with chemical reagents from Dinâmica Química (São Paulo, Brazil). Swine maxillae and esophagi were obtained from the Angelelli Ltd., a slaughterhouse in Piracicaba (São Paulo, Brazil), which was certified by the São Paulo State Department of Agriculture and Supply (SIF 2259). Permeation assays were performed in vertical transdermal Franz diffusion cells (Hanson Research Corporation, Chatsworth, CA, USA). Analyte quantification was performed using a high-performance liquid chromatography (HPLC) system coupled to an automatic collector and controlled with Surveyor ChromQuest 5.0 software (Thermo Scientific, Waltham, MA, USA).

### 2.2. Mucosa Preparation

Swine (*Sus scrofa domestica*, Landrace, animals aged 5 months and weighing around 75–80 kg) esophagi and maxillae were transported to the laboratory in PBS solution, within 30 min after slaughter. The esophageal and buccal epithelia were prepared according to the methods described by Diaz Del Consuelo et al. [13] and Franz-Montan et al. [21], respectively. In order avoid any risk of misidentifying the two epithelium types, they were handled on different days.

**Figure 1:**
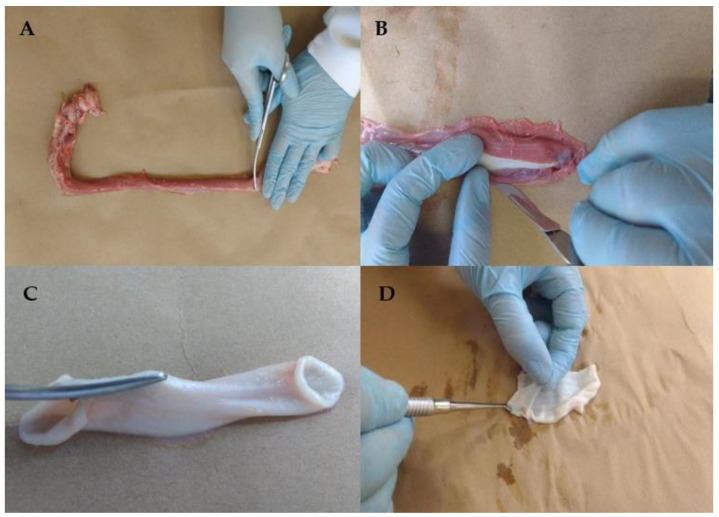
Esophagus mucosa preparation steps: (**A**) the ends of the esophagus were discarded; (**B**) the esophageal mucosa was gently separated from the external muscle layer with a scalpel, (**C**) the esophagi was opened longitudinally with scissors; (**D**) detachment of the epithelium from the connective tissue.

Briefly, about 3 cm from the ends of the esophagus were discarded (Figure 1A); the esophageal mucosa was gently separated from the external muscle layer with a scalpel (Figure 1B), and it was opened longitudinally with scissors (Figure 1C). The resulting mucosa piece was placed in a distilled water bath at 60 °C for 2 min, followed by careful detachment of the epithelium from the connective tissue (Figure 1D).

**Figure 2:**
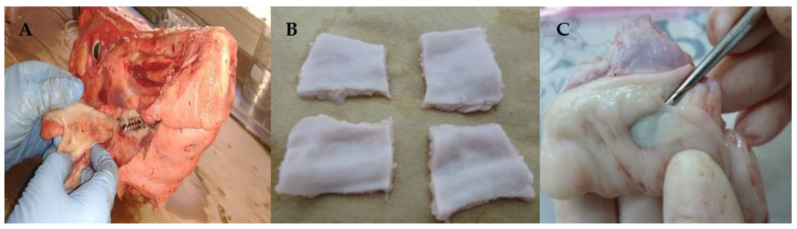
Buccal mucosa preparation steps: (**A**) the buccal mucosa (inner portion of the cheek) was isolated from the maxillae; (**B**) isolated buccal mucosa pieces; (**C**) detachment of the epithelium from the connective tissue.

The buccal mucosa (inner portion of the cheek) was isolated from the maxillae (Figure 2A,B) and immersed in distilled water at 60 °C for 2 min, prior to separation of the epithelium from the connective tissue (Figure 2C).

Each experiment was performed with tissues from at least three different animals. The buccal and esophageal mucosas were separated from the adjacent tissues using a scalpel blade and immersion in a distilled water bath (60 °C for 2 min). The epithelium was gently detached from the connective tissue (lamina propria) with a round spatula, followed by initial visual inspection of the epithelium samples to detect macroscopic tissue irregularities. Half of the specimens were used immediately after preparation (fresh tissue), while the remainder were stored for three weeks at −20 °C.

### 2.3. Histological Assessment

The fresh and frozen esophageal and buccal epithelium samples were examined to confirm the epithelium type and its integrity after the preparation and freezing processes.

The epithelium samples were fixed in 10% buffered formalin, dehydrated using a series of increasing ethanol concentrations (30%, 50%, 70%, and 100%), and subsequently embedded in paraffin and cut to 5 μm thicknesses using a microtome. Tissue samples were stained with hematoxylin–eosin prior to analysis using an optical microscope (Model DMLP, Leica Microsystems GmbH, Wetzlar, Germany) coupled to a digital color camera (Leica DFC280) [21].

### 2.4. Electrical Resistivity (ER) Measurement

After tissue preparation and initial visual inspection, ER assays were performed [22,23,28]. The resistivity measurements and the permeation assays employed a vertical Franz diffusion cell with permeation area of 1.77 cm^2^ and receptor compartment volume of 7.0 mL containing PBS at 37 °C. The epithelium was positioned in the Franz cell so that the basal layer was facing the receptor compartment.

The donor and receptor compartments were both filled with degassed PBS buffer and were fitted with Ag/AgCl electrodes connected to a Keysight 33220A signal generator (Agilent Technologies, Barueri, São Paulo, Brazil) and an ET-2053DMM digital multimeter (Minipa, São Paulo, Brazil), respectively. After allowing 1 h for electrolytic equilibrium under magnetic stirring (350 rpm) in a water bath at 37 °C, alternating current at 100 mV (rms) and frequency of 10 Hz was applied for resistivity evaluation. In order to avoid the effects of temperature, lipid composition, and barrier structural integrity [29], all the ER measurements were carried out using epithelium from animals of the same age and with very similar features of feeding and weight, at 37 °C. Finally, prior to each experiment, the macroscopic tissue integrity was checked for any mucosa perforations.

The electric current generated was measured by the multimeter in the receptor compartment, enabling the calculation of ER according to Ohm’s law (Equation (1)):

*ER* = (Δ*P*/*I*)/*A* (1)

where, Δ*P* is the potential difference of the system (mV), *I* is the measured current (µA), and *A* is the area (cm^2^).

Tissues exhibiting initial ER equal to or greater than 3 kΩ/cm^2^ were used in the subsequent stages of the experiment [28].

Specimens of the buccal and esophageal tissues (*n* = 20) were submitted to ER measurements. Following this, the permeation areas of half of the specimens of each tissue were delimited prior to freezing (Section 2.4). The remaining specimens were submitted to permeation assays (Section 2.5).

The frozen specimens were thawed for 15 min at room temperature, after which the ER measurements were repeated, followed by permeation assays (Section 2.5).

Following the permeation assays with fresh or frozen epithelium, the formulation was carefully removed, and fresh PBS buffer was added to the donor compartment. The permeation area of the tissues was intentionally damaged by piercing it five times using a 23-G hypodermic needle. These holes were not visible by observation. After this procedure, the ER was measured again (“Perforated”).

### 2.5. Tissue Storage

For storage, half of the specimens initially presenting ER greater than 3 kΩ/cm^2^ were wrapped in a cellulose filter (Unifil, 80 g/m^2^, Adria Laboratótrios, Londrina, Paraná, Brazil), moistened with PBS, and then wrapped in aluminum foil. The samples were packed in a sealed plastic bag and stored for three weeks in a freezer at a controlled temperature of −20 ± 1 °C.

### 2.6. Permeation Assays

Lidocaine hydrochloride (LDH) was the drug model used for the permeation assays. A 5% lidocaine hydrogel was prepared as described previously [21].

The experiments were performed with the fresh or frozen swine tissues, using a vertical Franz-type diffusion cell (Section 2.1). Immediately after the epithelial resistivity measurement, the buffer solution in the donor compartment was replaced, using 300 mg of lidocaine hydrogel, under occlusive and infinite dose conditions. Sink conditions were maintained throughout the experiment (LDH solubility in PBS: 0.24 ± 0.04 g/mL, determined by saturation of the model drug in PBS, prior to undertaking the permeation assays).

During 1 h permeation assays, the temperature and stirring conditions were the same as in the electrical resistivity assays. At 10 min intervals, aliquots of solution (300 μL) were withdrawn from the receptor compartment for analysis of LDH by HPLC (described below). The same volume of PBS was added to the receptor compartment, with a calculation of the dilution. After quantification by HPLC, individual graphs (for each vertical diffusion cell) were obtained by plotting the amount of LDH accumulated in the receptor compartment versus time (the intervals for each collection). The transport of LDH was analyzed according to a passive diffusion model, as commonly performed in studies of permeation across buccal and esophageal mucosas [13,14,30,31]. The steady-state flux (*J_ss_*) was mathematically calculated according to Fick’s first law (Equation (2)). This mathematical model is well known for use in the analysis of permeation assays and for comparisons of studies [32].

(2) $$J_{ss}=\;\frac{∆Qt}{\left(∆t\;\times \;A\right)}\;\left[\mu g/{\mathrm{cm}}^{2}/\min\right]$$

where ∆*Qt* is the difference of the amount of drug permeated, ∆*t* is the difference of the measurement time points (min), and *A* is the permeation area (cm^2^).

### 2.7. Lidocaine Analysis

Analysis of LDH was performed by HPLC, following a previously validated method with slight modifications [33]. Briefly, the mobile phase was a 60:40 (*v*/*v*) mixture of acetonitrile and 25 mM NH_4_OH, adjusted to pH 7.0 with H_3_PO_4_ solution, at a flow rate of 1.2 mL/min. A reversed phase column (150 × 4.60 mm, 5 μm, Phenomenex) was used, for which the injection volume was 20 µL, and the detector wavelength was 220 nm. The specificity of the method was checked using a triplicate calibration curve on three different days, with different concentrations of lidocaine solution. The limits of detection and quantification were 0.24 and 0.80 mg/mL, respectively.

### 2.8. Statistical Analysis

The D’Agostino–Pearson and Bartlett tests were used to assess data normality and the homoscedasticity of variances, respectively. The data for the amounts of LDH permeated over time were submitted to linear regression analysis. The resistivity and steady-state flux results were evaluated using Brown–Forsythe ANOVA and an unpaired *t*-test with Welch’s correction. The data for the cumulative amounts of LDH permeated after 1 h (*Q_1h_*) were submitted to ANOVA with Tukey’s test.

Correlations between ER and the steady-state flux, considering each storage condition and tissue, were analyzed using Pearson’s correlation (*rP*) test. All the statistical analyses were performed using GraphPad Prism 7.0 software (GraphPad Software, La Jolla, CA, United States), considering a significance level of 5%.

## 3. Results

### 3.1. Histological Controls

**Figure 3:**
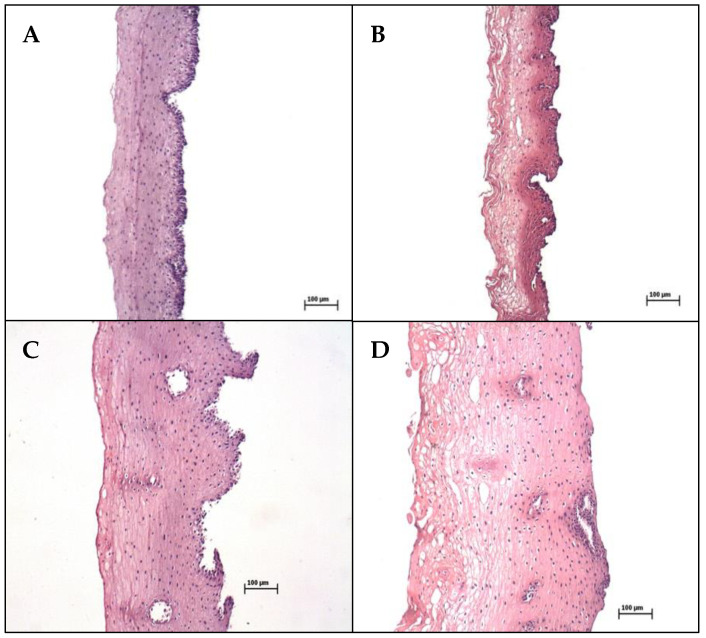
Histological sections of fresh (left panel) and frozen (right panel) epithelia from esophageal (upper images) (**A**,**B**) and buccal (lower images) (**C**,**D**) mucosas.

As shown in Figure 3, histological sections of fresh and frozen esophageal and buccal epithelia were successfully separated from the connective tissues. The samples presented intact morphology and integrity, confirming the presence of a stratified squamous epithelium, with tightly attached cells arranged in layers.

### 3.2. Electrical Resistivity

**Figure 4:**
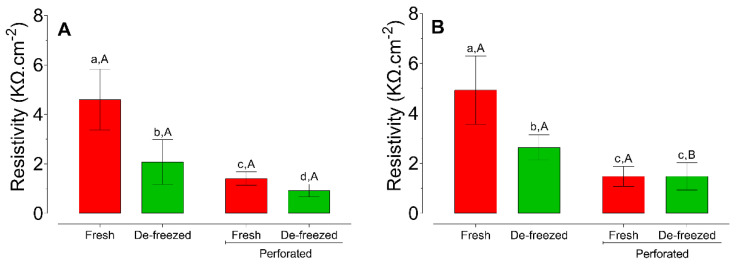
Mean (± SD) electrical resistivity values (kΩ/cm^2^) for the esophageal (**A**) and buccal (**B**) mucosas before and after freezing. Capital letters above the bars indicate statistically significant differences between the mucosas, considering the same storage conditions. Lowercase letters above the bars indicate statistically significant differences between the storage conditions, considering the same mucosa.

There was no difference between the ER values of the fresh esophageal and buccal epithelia (*p* = 0.4402). Similarly, there was no difference between the values for these epithelia after freezing (*p* = 0.11) or after perforation of the fresh epithelium (*p* = 0.6788). However, lower ER values were observed after perforation of the frozen esophageal epithelium (*p* = 0.0121), compared to the buccal epithelium in the same condition (Figure 4).

Lower values of ER were observed after freezing and after perforating compared to the initial values for both the esophageal epithelium (*p* < 0.0001 and *p* < 0.0001, respectively) and the buccal epithelium (*p* < 0.0001 and *p* < 0.0001, respectively) (Figure 4).

### 3.3. Permeation Assays

**Figure 5:**
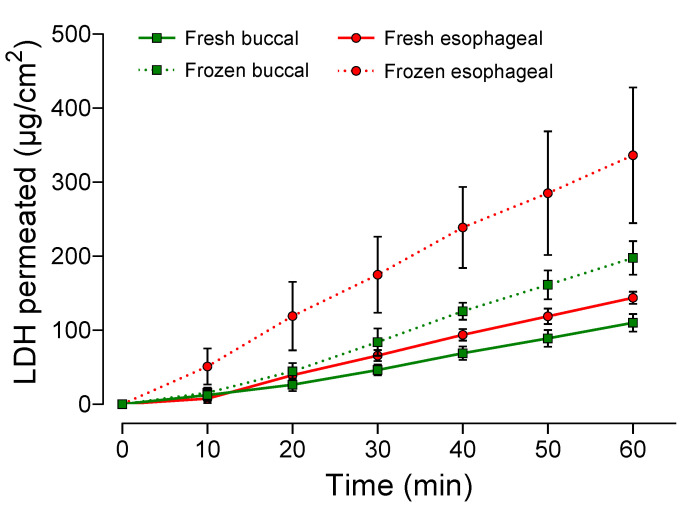
Profiles (mean ± SD, *n* = 9–10) for the permeation of lidocaine hydrochloride (LDH) during 1 h across fresh and frozen swine esophageal and buccal mucosas, for the formulation applied under infinite dose conditions. Linear regression analysis between curves: fresh buccal × esophageal (*p* = 0.0008); frozen buccal × esophageal (*p* < 0.0001); fresh buccal × frozen buccal (*p* < 0.0001); fresh esophageal × frozen esophageal (*p* < 0.0001).

The permeation of LDH showed linear profiles under infinite dose and occlusive conditions, with increases in the total amount of LDH permeated over time for both epithelia and conditions (Figure 5). Linear regression analysis showed that LDH presented higher permeation across the fresh (*p* = 0.0008) and frozen (*p* < 0.0001) esophageal epithelium, compared to the buccal epithelium. Higher permeation of LDH across both esophageal and buccal epithelia was observed after freezing, compared to the corresponding fresh tissues (*p* < 0.0001 and *p* < 0.000, respectively) (Figure 5).

The calculated permeation parameters are summarized in Table 1. The linear portions of the lines (angular coefficients) used for the steady-state flux (*J_ss_*) determinations were between 15 and 60 min in all cases. For both the fresh and frozen tissues, the permeation profile regression analysis showed that *J_ss_* and the total amount of LDH permeated (*Q_1h_*) were higher for the esophageal epithelium, compared to the buccal epithelium (*p* < 0.05 for all comparisons).

After freezing, there were significant increases of *J_ss_* and *Q_1h_* for both the esophageal epithelium (*p* < 0.05 for both parameters) and the buccal epithelium (*p* < 0.0001 for both parameters), with approximately two-fold higher LDH fluxes across the frozen tissues (Table 1).

### 3.4. Correlation Analysis

**Figure 6:**
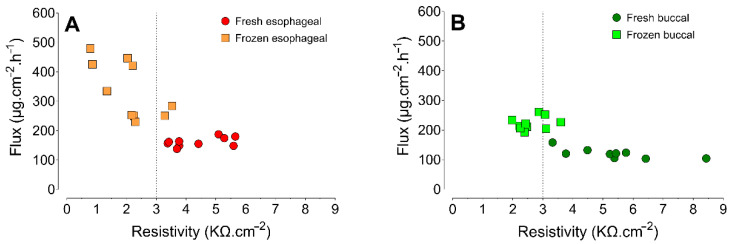
Correlation between steady-state flux and electrical resistivity, according to storage condition of (**A**) esophageal and (**B**) buccal epithelium.

Figure 6 shows the correlations between the ER values and the steady-state fluxes. No significant correlations between these variables were observed for the fresh esophageal mucosa (*rP* = 0.5, *p* = 0.1366) or the frozen buccal mucosa (*rP* = 0.29, *p* = 0.4102). However, the frozen esophageal mucosa (*rP* = −0.65, *p* = 0.0414) and the fresh buccal mucosa (*rP* = −0.74, *p* = 0.0215) showed strong inverse correlations between ER and the steady-state flux.

## 4. Discussion

This study investigated whether ER measurement could be used as an effective method to evaluate the integrity of esophageal and buccal epithelia employed in in vitro transmucosal permeation studies. It was decided to only use mucosal epithelium obtained by a heat-separation process, since this procedure has been demonstrated to have no impact on the morphology and permeability of oral cavity epithelium or esophagus mucosa [13,15,21]. Histological controls of the fresh heat-separated epithelia (Figure 3A,C) confirmed their intact structures, with stratified squamous, non-keratinized epithelium presenting tightly attached cells, separated from the connective tissue at the basal layer, in agreement with previous reports [14,15,21].

To assess the reliability of this method, first a comparison was made between the ER values for pre-selected intact tissues and intentionally damaged mucosas. The pre-selection of intact epithelia (ER > 3 kΩ/cm^2^) was based on previous findings, demonstrating that the permeation of hydrophilic drugs across esophageal epithelia remained the same for ER values between 2.8 and 12.7 kΩ/cm^2^ [28].

The perforated buccal and esophageal mucosas showed significantly lower ER values for both the fresh and frozen samples (Figure 3). This suggests that ER could detect invisibly damaged epithelium, under the conditions employed in the present study. The effect of physical damage on ER was observed previously for esophageal epithelium mucosa [28] and skin samples [25,34,35].

Cubayachi and colleagues [28] obtained ER values lower than 1.5 kΩ/cm^2^ for pierced samples, although the extent of damage could not be elucidated, since the number of holes and the damage procedure were not described. In the present study, a wide range of ER values was observed for damaged fresh and frozen esophageal mucosa (from 0.91 to 1.92 kΩ/cm^2^ and from 0.66 to 1.44 kΩ/cm^2^, respectively) and buccal mucosa (from 0.84 to 1.97 kΩ/cm^2^ and from 0.72 to 2.25 kΩ/cm^2^, respectively). It has been observed previously that the conductance values of damaged skin can also be quite variable, despite creating the same number of holes [25,34]. In the present study, the ER values for the intentionally damaged frozen tissues were significantly lower than for the intentionally damaged fresh tissues, suggesting that the physical damage caused by needle perforation was increased by the freezing process.

Analysis was also made of correlation between the steady-state fluxes of LDH across the fresh or frozen epithelium samples and the corresponding ER values prior to the permeation experiment. LDH was chosen as a model hydrophilic drug for the permeation assays, because it has been used in ER measurements of ion transport across the skin, involving a hydrophilic transport pathway [36,37,38]. Hence, in the present work, it was hypothesized that similar behavior would apply to the mucosa epithelium barrier.

The analysis of correlation between the steady-state flux and ER showed that when the fresh esophageal epithelium (ER ≥ 3 kΩ/cm^2^) was used as a barrier, the permeation of LDH was independent of ER, since no significant correlation was observed between these variables. However, when the same barrier was submitted to the freezing and storage process, there was a strong inverse correlation between ER and the steady-state flux of LDH, indicating that the permeation was influenced by ER. Similarly, previous work found that when ER was lower than 2.5 kΩ/cm^2^, greater amounts of lidocaine and prilocaine permeated across swine esophageal epithelium [28].

In the present study, the permeation of LDH increased for both frozen epithelia (Figure 5, Table 1), with mean ER values between 2.0 and 2.6 kΩ/cm^2^ obtained for the frozen esophageal and buccal epithelia, respectively (Figure 4). The histological images of the frozen specimens (Figure 3B,D) evidenced tissue damage after the freezing process (spherical spaces throughout the layers of the epithelium, with reduced contact among the cells), in agreement with previous reports [13,21], providing an explanation for the significant reductions in ER after freezing for both epithelium types (Figure 4). This has been observed previously for skin samples, which have presented reduced ER and increased permeability after freezing [35].

However, when fresh buccal mucosa was used, there was a strong inverse correlation between ER and the steady-state flux of LDH, suggesting that the permeation was influenced by ER and that a cut-off value of 3 kΩ/cm^2^ might not be sufficient to confirm the integrity of the epithelium from this region. Mastication comprises movements of the tongue, lips, and cheeks [39], all of which are regions are liable to injuries that could affect the integrity of fresh or frozen epithelium samples. Consequently, ER might not reflect permeation across the frozen epithelium, since the tissue was already damaged in the fresh condition. In fact, although the freezing process led to a decrease of ER and an increase of the steady-state flux of LDH (Figure 6, Table 1), no significant correlation was observed between ER and the flux across the frozen buccal epithelium.

Diaz Del Consuelo’s research group proposed the use of esophageal mucosa as a permeability barrier model, substituting buccal tissue, since the latter has a limited permeation area and damage caused by chewing [13,14,30]. It was shown that the esophageal epithelium presented comparable permeability characteristics for fentanyl citrate [13,14,30]. In the present study, linear regression analysis of the LDH permeation curve profiles showed a clear tendency for permeation to increase over time, with significantly increased permeation parameters when either fresh or frozen esophageal epithelium was used, compared to buccal epithelium (Figure 5, Table 1). Similar results were reported by Caon and Simões [19] for triamcinolone acetonide, but not for carbamazepine. These differences could be attributed to the different lipophilicities of the drugs used in the studies.

In the present work, despite the higher permeability of the esophageal epithelium compared to the buccal epithelium, there was no difference in ER between these two epithelia for either condition (Figure 4). The higher permeability of the esophageal epithelium could be attributed to its lower thickness, as shown in the histological images (Figure 3). Similarly, the greater thickness of buccal epithelium has been reported previously [13,15]. However, considering that ER is quite variable, in the present study this method was not able to detect the differences in permeability between the epithelia.

Nevertheless, there are conflicting reports regarding the effect of freezing and storage on the permeability of esophageal and buccal epithelial mucosas. The effect of freezing on the permeability of epithelial mucosa might be significant for some drugs [15,21,40], but might not influence the permeability for others [13,19,30].

Diaz del Consuelo and colleagues [13] found that for fentanyl citrate, the profiles for permeation across esophageal and buccal epithelial mucosas were not altered after up to three weeks of freezing (at −20 or −196 °C). Similarly, Caon and Simões [19] reported that freezing at −80 °C for no longer than a month did not affect the permeability of buccal or esophageal epithelia for acetonide triamcinolone and carbamazepine. These results suggest that the lipophilic nature and low molecular weight (<600 Da) of these drugs could have facilitated permeation by means of the transcellular route [10], so the permeabilities of the epithelia were not compromised by freezing.

However, changes in the permeation of buspirone, bupivacaine, antipyrine, and caffeine were observed after freezing buccal mucosa at −20 °C for periods longer than 24 h [15]. In earlier work, our research group demonstrated that freezing at −20 °C for up to 4 weeks led to increased permeation of LDH across epithelia from the buccal region or the dorsum of the tongue, but not across palatal epithelium [21]. Generally, the effects of epithelia freezing are greater for hydrophilic drugs, because the spaces created between the epithelial cells, as observed in histological sections of frozen epithelium specimens [13,21], could facilitate the transport of small hydrophilic species by means of paracellular (intercellular) permeation [7,8]. It has been suggested previously that the buccal mucosa acts as a stronger barrier to the diffusion of hydrophilic drugs, compared to lipophilic compounds [41]. In addition, the type of epithelial mucosa (such as keratinized or not) and the freezing conditions also seem to contribute to the permeability.

It should be noted that in all the studies cited, the integrity of the barrier was only evaluated by means of histological images and permeability measurements. The ER method has been widely used to assess skin integrity [22,23,24,25,26,35], but only a few studies have used it to evaluate mucosa integrity [27,28,42,43]. However, due to the differences between studies in terms of mucosa preparation, such as the use of full-thickness dermatome mucosa [27,43] or heat-separated epithelium [28,42], there is still a lack of information regarding ER cut-off values for the selection of intact esophageal or buccal epithelia for use in in vitro permeation studies using vertical diffusion cells.

## 5. Conclusions

The results of the present study suggested that ER measurement could provide reliable prediction of tissue integrity for mucosa epithelium, as previously found for skin. For esophageal mucosa epithelium (but not for buccal mucosa epithelium), ER ≥ 3 kΩ/cm^2^ could be used as an exclusion criterion for tissue samples, prior to in vitro permeation studies. However, the use of esophageal epithelium might overestimate the absorption of hydrophilic drugs.

Additionally, freezing and storage may have a significant effect in relation to tissue damage. Therefore, it would be preferable to determine the permeability of hydrophilic compounds using fresh tissues.

## Figures and Tables

**Table 1:** Calculated steady-state fluxes (*J_ss_*) and cumulative amounts of LDH permeated after 1 h (*Q_1h_*) for permeation across the different mucosas, under infinite dose conditions (*n* = 9–10; *R*^2^ > 0.98). * EE: enhancement effect comparing the mean *J_ss_* values for the fresh and frozen tissues.

Tissue		*J_ss_* (µg.cm^−2^.h^−1^)	EE *	*Q_1h_* (µg)
Esophageal	Fresh	161.6 ± 15.4 ^a^	----	254.3 ± 14.7 ^a^
	Frozen	337.4 ± 96.3 ^b^	2.08	595.0 ± 162.3 ^b^
Buccal	Fresh	120.9 ± 17.0 ^c^	----	194.8 ± 21.0 ^c^
	Frozen	222.3 ± 21.7 ^d^	1.83	349.7 ± 40.1 ^d^

Application of ANOVA/Tukey’s test for the steady-state flux (*J_ss_*, mean ± SD) of LDH through fresh and frozen esophageal and buccal swine mucosas: fresh esophageal × fresh buccal (*p* < 0.0001); frozen esophageal × frozen buccal (*p* = 0.0043); fresh esophageal × frozen esophageal (*p* = 0.0002); fresh buccal × frozen buccal (*p* < 0.0001). Application of ANOVA/Tukey’s test for the cumulative amounts of LDH permeated after 1 h (*Q_1h_*, mean ± SD) through fresh and frozen esophageal and buccal swine mucosas; *p* < 0.0001 for all comparisons. Different superscript letters in the same column indicate statistically significant differences.

## Data Availability

The data presented in this study are available on request from the corresponding author.
